# Colitis Cystica Profunda of the Hepatic Flexure: A Case Report

**DOI:** 10.7759/cureus.58342

**Published:** 2024-04-15

**Authors:** Ryan Denis, Mikayla Hobbs, Martin Felix, Henry J Lujan

**Affiliations:** 1 General and Colorectal Surgery, St. George's University School of Medicine, St. George, GRD; 2 Internal Medicine, St. George's University School of Medicine, St. George, GRD; 3 Colorectal Surgery, Jackson Memorial Hospital, Miami, USA

**Keywords:** colitis cystica profunda in the ascending colon, surgery general, surgical pathology, right colon cancer, colitis cystica profunda

## Abstract

A 72-year-old woman with a prior sigmoid resection for colon cancer underwent a right hemicolectomy after a colonoscopy revealed a mass in the hepatic flexure. A preoperative biopsy at colonoscopy showed tubulovillous dysplasia with high-grade neoplasm. The final specimen pathology revealed benign mucosal elements with mucin pools consistent with colitis cystica profunda (CCP). CCP is a benign lesion; no further treatment was necessary after resection. To our knowledge, this is the first reported case of CCP in the right colon, presenting atypically in the hepatic flexure. This case report brings to light the difficulty and importance of making an accurate diagnosis of CCP.

## Introduction

Colitis cystica profunda (CCP) is a rare benign non-neoplastic disease with an estimated prevalence of approximately one in 100,000 persons per year [1]. It occurs predominantly in the rectum and sigmoid colon and is rarely seen in the ascending colon, small intestines, and stomach. Pathologically, CCP manifests as mucus-containing cysts of varying sizes and morphology that spread into the submucosa, muscularis propria, and even the serosa. The specific lesion in our patient extended into the muscular layer of the hepatic flexure. The pathogenesis of CCP is currently unknown, but it is believed to be attributable to congenital or acquired mucosal muscle weakness. CCP can occur at any age but is more commonly found in middle-aged men 30-40 years old, with clinical manifestations being very nonspecific. Symptoms such as diarrhea, abdominal pain, blood and mucus in stool, constipation, urgency, and rectal pain are common presentations. There are two main types of CCP: the diffuse type and the local type. Diffuse CCP is commonly found in the left colon, while the local type presents more commonly in the rectum. Some studies have also revealed that 40% of patients present with multiple ulcers, 20% present with a singular ulcer, and the rest of the lesions vary in shape and size [1]. Because CCP has gross and microscopic characteristics similar to mucinous adenocarcinoma, it is important to get accurate readings of microscopic findings [2,3]. Due to the limited number of reported cases, including the only reported case of CCP in the right colon to our knowledge, proper recognition and diagnosis of this disease are rare. Our case report aims to increase understanding and awareness of this disease.

## Case presentation

**Figure 1:**
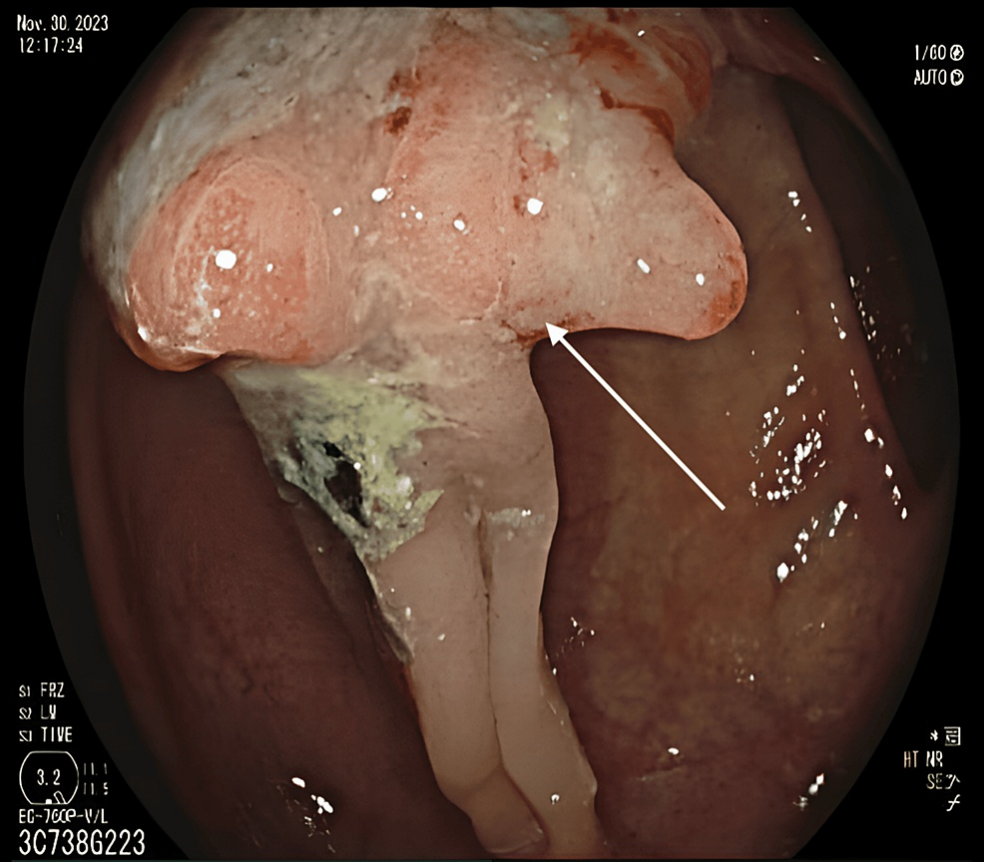
The colonoscopy image shows a large fungating ulcerated polyp at the hepatic flexure.

A 72-year-old woman with a past medical history of T2N0 colon cancer with a sigmoid colectomy three years prior presented for a surveillance colonoscopy with her gastroenterologist. The patient was asymptomatic at the time of her colonoscopy. The gastroenterologist identified and tattooed a hepatic flexure tubulovillous high-grade neoplasm (Figure 1), and the patient was referred for surgical consultation. The surgical team advised a robotic right hemicolectomy. At the time of surgery, the surgical team visualized the tattoo in the proximal transverse colon and performed a robotic extended right hemicolectomy.

**Figure 2:**
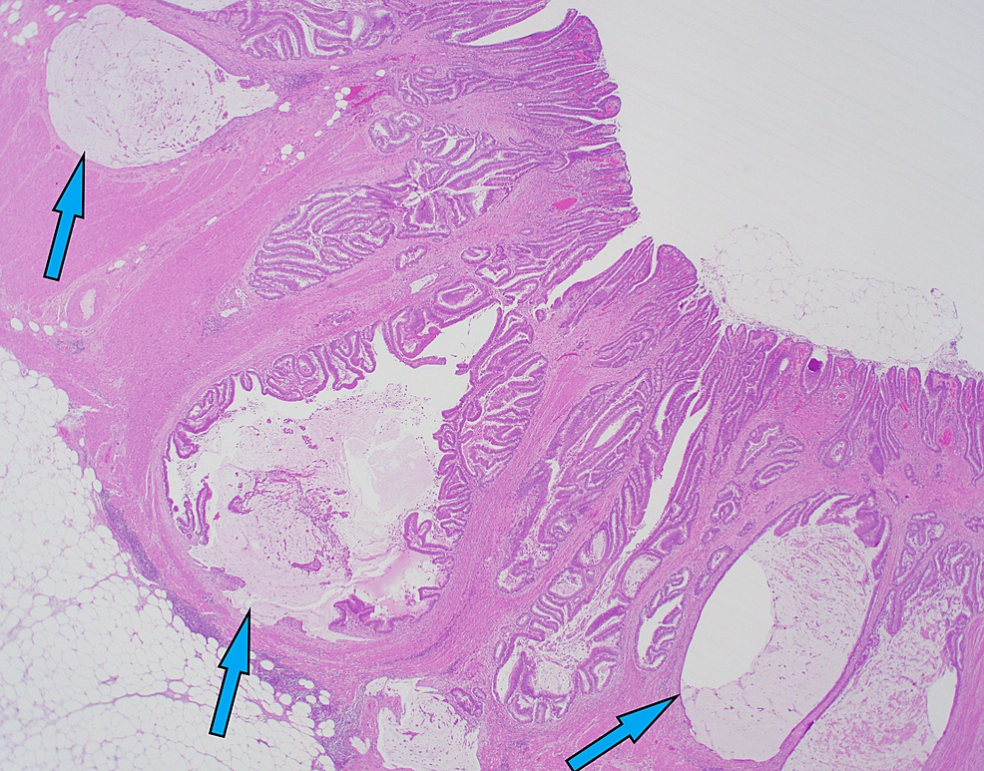
Histopathologic image displaying rounded aggregates of benign mucosal elements and mucin pools (hematoxylin and eosin).

The pathology of the resected specimen reported a 2.5 x 2.5 x 0.8 cm polypoid mass with an ulcer in the ascending colon located 11 cm from the distal margin, 19 cm from the proximal margin, and 10 cm from the radial margin. The tumor grossly extended up to the muscularis propria. The polyp consisted of a large tubulovillous adenoma, along with rounded aggregates of benign mucosal elements and mucin pools in the colonic wall consistent with CCP (Figure 2). The findings, which include ulcers, hemosiderin deposits, and inflammation, point to trauma-induced displacement rather than invasive adenocarcinoma. The immunostain showed preserved MLH1, MSH2, PMS2, and MSH6. These results do not suggest the presence of DNA mismatch repair deficiency in the adenoma. Additionally, 16 lymph nodes were harvested, and all were benign. As the diagnosis of CCP is rare, we confirmed the pathology with a second independent pathologist.

## Discussion

CCP is a rare and benign lesion of the colon characterized by the presence of cysts of varying sizes in the submucosa, lined by mucin-secreting columnar epithelium extending deep into the colonic wall [4]. Although it can manifest within the entire gastrointestinal system, CCP predominantly presents in the rectum and sigmoid colon and less frequently in the stomach and small intestine. Our patient presented with a mass in the distal ascending colon near the hepatic flexure. The presentation in this location makes this type of CCP significantly uncommon and, to our knowledge, the only documented case. This condition frequently mimics malignant processes. Therefore, it is paramount to distinguish it from conditions such as mucus-producing adenocarcinoma.

The pathogenesis of CCP is also unknown and is thought to be due to either congenital or acquired etiologies. Embryological studies and pediatric case reports support the former. The latter is supported by associations with conditions that irritate the bowel wall, such as inflammatory bowel disease, and experimental studies implicating an inflammatory etiology [5]. Diagnosing CCP involves a combination of noninvasive (ultrasound, MRI, and CT scans) and invasive imaging (endoscopy, colonoscopy, and barium enema) with confirmation by biopsy. Invasive imaging cannot provide characteristic signs that distinguish CCP from other carcinomas [6]. On CT, CCP may appear as a non-infiltrating entity in the submucosa, while MRI will further reveal the mucoprotein content of the cysts [6].

CCP is most commonly diagnosed in the rectum. With rectal presentation, it is essential to make the diagnosis preoperatively to avoid unnecessary rectal resection of a benign lesion with the associated morbidity and mortality. In contrast, when presenting elsewhere in the colon, though imaging may be helpful if the diagnosis of CCP cannot be confirmed with endoscopic biopsies, surgical resection may still be indicated.

CCP may present with nonspecific symptoms such as abdominal pain, hematochezia, diarrhea, mucus in the stools (mucorrhea), and tenesmus [7]. However, many cases are asymptomatic and incidentally discovered during screening colonoscopy, as was the case with our patient. Treatment of CCP may vary from case to case. In symptomatic cases, treatment is directed at reducing these symptoms with conservative therapies before surgery. These conservative therapies consist of diet and lifestyle modifications and pharmacologic treatment. Diet and lifestyle modifications include a diet high in fiber, lubricants, and bulk laxatives, while pharmacologic therapies may include docusate sodium, hydrocortisone enemas, and sucralfate suspensions [8]. If symptoms persist, surgery with resection of the mass is the recommended treatment. When feasible, complete excision by snare polypectomy or endoscopic mucosal resection may be used to avoid more invasive procedures such as colectomy or hemicolectomy. Due to the coincidental discovery of the mass in our patient and its highly unusual location for CCP on surveillance colonoscopy, along with the propensity of the mass to be malignant and the past medical history of sigmoid colon cancer, conservative treatment was omitted, and surgical resection with hemicolectomy was the primary plan of care.

## Conclusions

CCP is a benign lesion characterized by intramural or submucosal mucous-containing cysts that mimic the presentation of colonic adenocarcinoma. Patients typically experience nonspecific symptoms, such as blood in the stool, tenesmus, and diarrhea, in association with the lesion when it presents in the rectum. Considering our patient's previous medical history of colon cancer and no preoperative diagnosis of CCP, she was advised to undergo a right hemicolectomy when a tubulovillous adenoma was discovered on routine colonoscopy. The pathology post-resection subsequently led to the diagnosis of CCP. Our case demonstrates the challenge of preoperatively diagnosing CCP when it is not present in the rectum.
